# A New Polysaccharide Carrier Isolated from Camelina Cake: Structural Characterization, Rheological Behavior, and Its Influence on Purple Corn Cob Extract’s Bioaccessibility

**DOI:** 10.3390/foods11121736

**Published:** 2022-06-14

**Authors:** Lucia Ferron, Chiara Milanese, Raffaella Colombo, Raffaele Pugliese, Adele Papetti

**Affiliations:** 1 Department of Drug Sciences, University of Pavia, 27100 Pavia, Italy; lucia.ferron01@universitadipavia.it (L.F.); raffaella.colombo@unipv.it (R.C.); 2 FlaNat Research Italia Srl, 20017 Rho, Italy; 3 C.S.G.I. & Department of Chemistry, Physical Chemistry Section, University of Pavia, 27100 Pavia, Italy; chiara.milanese@unpv.it; 4 NeMO Lab, ASST Grande Ospedale Metropolitano Niguarda, 20162 Milan, Italy; raffaele.pugliese@nemolab.it

**Keywords:** camelina cake polysaccharide, carrier, stabilizer, purple corn cob, by-product, anthocyanin bioaccessibility

## Abstract

A polysaccharide fraction obtained from camelina cake (CCP), selected as a carrier to encapsulate purple corn cob extract (MCE), was investigated. A wide population of carbohydrate polymers (with a polydispersivity index of 3.26 ± 0.07 and an average molecular weight of about 139.749 × 103 ± 4.392 × 10^3^ g/mol) with a gel-like behavior and a thixotropic feature characterized the fraction. MCE-CCP combinations (50–50 and 25–75, *w*/*w*), selected based on CCP encapsulation efficiency, were tested for their stability and MCE polyphenols’ bioaccessibility during digestion (monitored using an in vitro static procedure). During the oral and gastric phases of the digestion process, CCP gradually swelled and totally released MCE polyphenols. MCE-CCP50 had the fastest release. Moreover, anthocyanins were still detectable during the duodenal phase, in both MCE-CCP ingredients. Furthermore, CCP (5 mg/mL) exerted in vitro potential hypocholesterolemic activity via bile salts binding during digestion.

## 1. Introduction

Nowadays, the agro-food industry generates a huge amount of waste, which constitutes one of the main environmental problems due to the high organic charge [1], but it could represent a potential source of valuable bioactive compounds, suitable for food, pharmaceutical, and textile industries [1,2]. Recently, the search for new and renewable sources of bioactive compounds has been encouraged by the European Commission, which targeted the achievement of a fully circular economy by 2050, also through the farm to fork strategy. This program represents the heart of the European green deal, which aims to achieve healthier and more environmentally friendly food systems [3].

Therefore, great effort has been made by scientists and companies in order to investigate and characterize the potential applications of agro-food by-product bioactive compounds or metabolites in food, pharmaceutical, and cosmetic industries [1,2]. These natural compounds can be used as both nutraceutical ingredients and additives thanks to their strong antioxidant capacities, and are thus potentially useful for the development of products with enhanced nutritional value, potential health benefits, and long shelf-lives [1].

Among agro-food wastes, corn cobs are the main by-products generated during corn processing, and purple corn cobs are still lacking added-value applications [4]. Purple corns (*Zea mays* L.) are pigmented corn varieties from South America, mainly Peru and Bolivia, which have strong antioxidant properties thanks to their high anthocyanin content [5]. Several studies have investigated the anthocyanin contents in purple corn extracts obtained from different tissues, i.e., kernel, cob, husk, and silk, and tested their bioprotective effects [6,7]. Husk and cob extracts are richer in anthocyanins than kernel extracts. Contents range from 0.49 to 4.6% and from 1.25 to 13.18% (*w*/*w* dry material) for cob and husk, respectively [7]; moreover, the husk and cob differ from the kernel by the presence of perlagonidin in their phytocomplex [8]. Moradyn is a new Italian purple corn variety obtained from a Peruvian corn line (Morado), and it is characterized by highly pigmented cobs. The extract obtained from Moradyn cob (MCE) is characterized by a phytocomplex rich in antioxidant compounds, such as anthocyanins, quercetin, and kaempferol derivatives, but a preliminary study highlighted that the bioaccessibility of these MCE polyphenols was markedly affected by digestion, leading to a decrease in bioactivity [8], as already reported for other natural extracts [9]; this behavior limits polyphenols bioavailability and their subsequent efficacy.

During the last five years, there has been growing interest in the application of encapsulation technology in order to entrap antioxidant compounds, protect them against environmental conditions during storage, extend their shelf lives, and enhance their bioavailability [10,11]. Different carriers have been employed, depending on the type and nature of core materials and on the targeted applications of the microencapsulated ingredients [10]. Several authors reported that vegetable polysaccharides such as Arabic gum, maltodextrins, inulin, and other purified native gums are carriers more stable, biocompatible, biodegradable, and versatile than proteins (which might be melted or denatured), since they better resist high temperatures (>40 °C) and pH changes [12]. In fact, their functional groups make them extremely versatile, as they can interact with a wide range of both hydrophilic and hydrophobic bioactive compounds. Examples are anthocyanin and quercetin derivatives which are consistent with water-based gel formulations, including gum, maltodextrin, and starch [13]. Moreover, polysaccharides are efficient at entrapping bioactives due to their high molecular weights and the presence of numerous functional groups in their structures [12]. In addition, among natural polysaccharides, gums have remarkable and specific rheological features, which have been strictly related to their biological properties and technological applications [5,14].

In our previous work, the polysaccharide fraction isolated from camelina cake (*Camelina sativa* L. Krantz) was selected as potential carrier for MCE, based on the high encapsulation efficiency values registered at 1:1 and 1:3 core/wall material ratios. Camelina sativa is an important seed oil crop belonging to the Brassicaceae family. It is cultivated worldwide, and its oil is a valuable and well-characterized source of unsaturated fatty acids, mainly linolenic and α-linoleic acids [15].

To the best of our knowledge, the potential application in the food industry of Camelina sativa’s by-product has never been investigated, so the use of camelina cake polysaccharide fraction (CCP) as a carrier for MCE could represent a valuable technological innovation. Preliminary spectroscopic analysis performed both on CCP and on MCE encapsulated with CCP indicated that CCP is mainly characterized by mannose, arabinose, and rhanmose residues, and by the presence of a protein component with a random coil conformation, suggesting a gum-like composition [16] maintained after MCE addition. CCP also increased MCE shelf-life when used as a carrier at 50 and 75% *w*/*w* [17].

Therefore, considering these preliminary results, the aim of the present work was to determine the CCP’s average molecular weight and rheological properties, since these physico-chemical and structural parameters are strictly related to the biological behavior of natural polysaccharides [16,18,19]. The relationship between the CPP’s physico-chemical features and its stabilizing properties was evaluated by submitting a MCE-based ingredient to simulated gastrointestinal conditions (applying a slightly modified INFOGEST protocol) [20] and monitoring the MCE bioaccessibility index for thirteen selected markers. CCP hypocholesterolemic activity was assessed by testing its bile salt binding activity [21].

## 2. Materials and Methods

### 2.1. Chemicals

Ethanol (96% *v*/*v*), methanol, HPLC-grade acetonitrile, phosphoric acid (85% *w*/*v*), and sodium chloride were obtained from Carlo Erba (Milan, Italy). HPLC-grade formic acid, hydrochloric acid (37% *w*/*v*), Type VI-porcine pancreatic α-amylase, pepsin from porcine gastric mucosa (≥400 U mg^−1^), bile extract porcine, pancreatin (8 × USP) from porcine pancreas, sodium taurocholate (NaTC), sodium glycocholate (NaGC), sodium taurodeoxycholate (NaTCDC), sodium glycochenoxycholate (NaGCDC), protease from Streptomyces griseus type XIV (≥3.5 U mg^−1^), viscozyme L cellulolytic enzyme mixture, sodium azide, and cholesterolamine were provided by Merck KGaA (Darmstadt, Germany).

Water was obtained from a Millipore Direct-QTM system (Merck-Millipore, Milan, Italy).

Pullulan gel filtration chromatography standard kit was purchased from Waters Corporation (Milford, MA, USA).

### 2.2. Camelina Cake Polysaccharide (CCP) Extraction

Camelina cake was kindly provided by FlaNat Research Italia S.r.l. (Milan, Italy).

A CCP dried extract was prepared following the procedure previously optimized [17]. Briefly, the cake obtained from cold-press oil production was immediately soaked with water (1:10 solvent/raw material ratio) and extracted at 115 °C for 15 min. After filtration of the supernatant, CCP was precipitated by adding a 96% ethanol solution (1:2.5, *v*/*v*) at 4 °C overnight. Finally, the polysaccharide fraction was separated by centrifugation at 5000 rpm (Neya 8 ZFKN-39276, Remi Eletrotechnik LTD, Mumbai, India) for 10 min at 25 °C, freeze dried (Modulyo freeze-drier s/n 5101, 5 Pascal, Trezzano sul Naviglio, Italy), and then used in the experiments.

### 2.3. Moradyn Cob Extract (MCE) Preparation

Moradyn chopped cobs were kindly provided by FlaNat Research Italia S.r.l. (Milan, Italy) and extracted with 50% aqueous ethanol for 3 h at 50 °C. The extract (MCE) was filtered through 0.45 µm membrane filters (Merck-Millipore, Milan, Italy) and the organic solvent removed under reduced pressure at 40 °C (Buchi R-II, Büchi Labortechnik AG, Flawil, Switzerland) [8]. Finally, MCE was freeze-dried (Modulyo freeze-drier s/n 5101, 5 Pascal, Trezzano sul Naviglio, Italy) and used in the experiments.

### 2.4. Preparation of MCE-CCP Ingredients

MCE was encapsulated in 50 or 75% CCP (*w*/*w*) to obtain MCE-CCP ingredients as follows: freeze-dried CCP was dissolved in water at 10 mg/mL final concentration and hydrated at 4 °C overnight. The carrier solution and MCE (core) were mixed at 1:1 or 1:3 ratio (*w*/*w*), and the obtained ingredients were dried under vacuum in a drying oven (42 °C, 8 mbar) for 48 h (Goldbrunn 1400, Expondo, Berlin, Germany) [17].

### 2.5. Mechanical Testing

The CCP rheological properties were investigated using a Kinexus DSR Rheometer (Netzsch, Selb, Germany) equipped with a parallel-plate geometry (acrylic diameter 20 mm; gap 34 μm). CCP viscosity was measured using a flow step program, at increasing shear rate (0.001–1000 s^−1^), to investigate their non-Newtonian behavior. To evaluate the storage (G’) and loss (G”) moduli of CCP, frequency sweep experiment results were recorded as a function of angular frequency (0.1–100 Hz) at 0.5% fixed strain. To test CCP thixotropy, shear-thinning tests were performed by a series of peak hold tests in which shear rates were kept constant, as previously reported by Pugliese et al., (2021) [22]. Briefly, a shear rate of 0.01 s^−1^ and a shear rate of 5.3 s^−1^ were applied in sequence for 60 and 20 s, respectively. Subsequently, a high shear rate of 1000 s^−1^ for 20 s followed by a shear rate of 5.3 s^−1^ for 20 s were applied. Lastly, a shear rate of 0.01 s^−1^ was used to simulate the shear condition of CCP at rest. Each experiment was performed in triplicate, and data were processed using OriginLabTM 8 software.

### 2.6. CCP Average Molecular Mass Determination

The CCP average molecular weight (Mw) and polydispersivity index (Pi) were deter-mined by size exclusion chromatography (SEC) using an Agilent 1200 chromatographic system coupled with a G7162A Refractive Index Detector (RID) (Agilent Technologies, Santa Clara, CA, USA). Narrow pullulan standards in the 5−642 × 10^3^ g/mol range were used for the calibration curve (Waters Corporation, Milford, MA, USA). An Ultrahydrogel™ 2000 column (12 µm, 7.8 mm × 300 mm) and an Ultrahydrogel™ 250 column (6 µm, 7.8 mm × 300 mm) (Waters Corporation, Milford, MA, USA) were coupled in series and operated at a constant flow rate of 0.8 mL/min. The mobile phase consisted of a 0.1 M NaCl solution (*w*/*v*) containing 0.02% NaN_3_ (*w*/*v*), filtered through a 0.45 µm PTFE membrane (Merck-Millipore, Milan, Italy). Columns and detector were maintained at 40 °C.

Samples were prepared by dissolving CCP or pullulan standards in 0.1 M NaCl (1 mg/mL final concentration) and filtered (cellulose microporous membrane filter, 0.45 mm, Merck-Millipore, Milan, Italy) before injection (injection volume 50 µL).

### 2.7. Determination of Total Polyphenol Content in CCP-MCE Ingredients by RP-HPLC-WVD Analysis

The encapsulated polyphenols were extracted following a procedure described by Norkaew et al. (2019) [23] with slight modifications: 5 mg of dried ingredients was dissolved in 1 mL deionized water, mixed, and then sonicated for 20 min; a methanol/acetonitrile/formic acid (60:35:5, *v*/*v*/*v*) mixture was added to obtain a 10 mL final volume. Samples were concentrated up to 0.25 mL under reduced pressure at 40 °C (Buchi R-II, Büchi Labortechnik AG, Flawil, Switzerland) and then diluted to a 5 mL final volume by means of a binary mixture consisting of 0.1% formic acid aqueous solution and 0.01% formic acid in acetonitrile 80:20 (*v*/*v*) before HPLC analysis.

Analyses were carried out using a 1260 Infinity II technology series system (Agilent Technologies, Santa Clara, CA, USA), equipped with a quaternary gradient pump, a vial sampler, a degasser, a thermostatted column system set at 25.0 ± 0.5 °C, and a variable wavelength detector (VWD). The HPLC-VWD system was controlled using Agilent OpenLab CDS ChemStation software—Windows 10. The chromatographic separation was carried out on a Gemini^®^ C18 analytical column (150 × 2.0 mm i.d., 5 μm, Phenomenex, Torrance, CA, USA) operating at 0.3 mL/min constant flow rate (injection volume 20 μL), using the mobile phase and the gradient elution program already applied and validated by Ferron et al. (2021) [24]. Chromatograms were recorded at 520 and 370 nm. The selected marker compounds’ identification was based on co-cromatography with analytical standards (when commercially available) and comparison with literature data [8]. The peak area (mAU) registered for the undigested compound was used to calculate the bioaccessibility index.

### 2.8. In Vitro Digestion Procedure

MCE-CCP 50% or MCE-CCP 75% water solutions (1 mg/mL) were submitted to an in vitro gastrointestinal simulation digestion process following the INFOGEST protocol; briefly, simulated salivary (SSF), gastric (SGF), and intestinal (SIF) fluids were prepared using proper mixtures of electrolytes, bile salts, water, and enzymes. Pepsin and pancreatin were directly added to SGF and SIF, respectively [20]. Changes occurring in phytocomplex composition were monitored by collecting 2.5 mL of sample directly from the flask at different time points during each digestion step (after 2 min for the oral phase; after 15, 30, 60, and 120 min for the gastric phase; and after 30 and 120 min for the intestinal phase). At the end of each monitored time, enzymes were inactivated (90 °C, 5 min) and samples were centrifuged (30 min, 4 °C, 5000 rpm) (Centrifuge 5804 R Eppendorf, Hamburg, Germany). Supernatants were freeze-dried (Modulyo freeze-drier s/n 5101, 5 Pascal, Trezzano sul Naviglio, Italy) and stored at −20 °C until analyses.

### 2.9. Bioaccessibility Evaluation

The percentage of soluble polyphenols in each collected digested sample represented the bioaccessible MCE fraction available for absorption.

The samples collected during the digestion process were dissolved in 2.5 mL of 0.1% formic acid aqueous solution—0.01% formic acid in acetonitrile (80:20, *v*/*v*)—and filtered through 0.2 µm nylon syringe filters (Phenomenex, Torrance, CA, USA) before HPLC analysis.

The bioaccessibility index for each monitored polyphenolic compound was calculated as:

(1) $$\mathrm{Bioaccessibility}\;\mathrm{index}\;(\%)=A_{dig}/A_{tot\;}\times 100$$

where *A_dig_* corresponds to the peak area (mAU) of the marker compound in the ingredients after digestion, and *A_tot_* represents the peak area (mAU) of the marker in the undigested sample, considered as 100%.

### 2.10. CCP Bile Salts Binding Capacity

The CCP bile salts’ binding capacity was evaluated following the protocol reported by Lin et al. (2020) [21], with some modifications. The procedure basically involved following three steps: CCP in vitro digestion in the presence of bile salts (BS), collection of free BS by centrifugation, and their quantification by RP-HPLC.

Five different CCP concentration levels (0.5, 0.75, 2.5, 5, 10 mg/mL) were tested, and cholestyramine (10 mg/mL) was used as a positive control [25]. Water was used as blank in the digestion process.

The in vitro digestion procedure was carried out following the standardized INFOGEST protocol, but reproducing the intestinal phase conditions with a 10 mM BS mixture (35% NaTC, 35% NaGC, 15% NaTCDC and 15% NaGCDC) instead of commercial bile, in order to mimic the BS composition and concentration typically present in an adult intestine under the fed condition. Samples collected at the end of the intestinal phase were centrifuged at 14,000 rpm for 30 min at 4 °C (Centrifuge 5804 R Eppendorf, Hamburg, Germany); subsequently the supernatants were filtered (0.2 µm nylon syringe filters, Phenomenex, Torrance, CA, USA) and immediately submitted to RP-HPLC analysis.

BS separation and quantification were performed by HPLC-WVD (1260 Infinity II system Agilent Technologies, Santa Clara, CA, USA), using a Zorbax SB-C18 column (150 mm × 4.6 i.d., 5µm, Agilent Technologies, Santa Clara, CA, USA) operating at 0.8 mL/min constant flow rate (injection volume 100 mL). The mobile phase consisted of 0.3 M phosphoric acid (solvent A) and acetonitrile (solvent B) with the following gradient table: 0–1 min, 25% B; 1–10 min, 25–43% B; 10–12 min, 43–44% B; 12–22 min, 44–90% B; 22–24 min, 90–25% B, and 10 min column reconditioning. Chromatograms were recorded at 200 nm.

A BS 10 mM mixture prepared dissolving NaTC, NaGC, NaTCDC, and NaGCDC in SIF was used to assess the separation efficiency of the HPLC method. To quantify the un-bound BS, a five-point standard curve was prepared for each BS in the concentration range 1–10 mM.

The binding activity was calculated according to Equation (2):

(2) Binding Activity (%)=[(BS blank−BS unbound)/BS blank]×100

where *BS blank* is BS total concentration (expressed in mM) registered after the water digestion process and *BS unbound* is BS concentration (expressed in mM) detected in supernatants after the CCP intestinal digestion phase.

### 2.11. Statistical Analysis

Statistical analysis of the data was performed using Microsoft Excel (version 365). The significant differences (*p* < 0.05) were evaluated by variance analysis (ANOVA). Experiments were performed at least in three replicates.

## 3. Results and Discussion

### 3.1. CCP Molecular Parameters

Considering that the average molecular weight and the molecular weight distribution affect the polysaccharide rheological and functional features [26], these parameters were extrapolated for camelina cake polysaccharides following the slice method reported by Garcia-Lopera et al. (2005) [27], based on data obtained from a SEC-RID system calibrated with pullulans (external standard method).

The registered chromatogram clearly indicated the presence of a varied population of carbohydrate polymers (number average molecular weight, Mn: 3.234 × 10^3^ g/mol) eluting in the range 17–23 min and representing about 100% of the eluted material. CCP molecular distribution weight was extrapolated from the pullulan standard calibration curve, and it was in the range 7.224 × 103−698.297 × 10^3^ g/mol. The average Mw was about 139.749 × 10^3^ ± 4.392 × 10^3^ g/mol; the Pi (Mn/Mw) was 3.26 ± 0.066.

These data and the results previously obtained by Fourier-Transform Infrared Spectroscopy (FT-IR) analysis [17] indicated that CCP Mw and Mn values were far lower than those reported for flaxseed (*Linum usitatis-simum* L.) mucilage, which had a composition and rheological behavior close to those of camelina seed mucilage [15]; however, CCP features were very similar to those obtained for a neutral polysaccharide fraction isolated from flaxseed mucilage. Therefore, we could hypothesize that CCP represented the neutral polysaccharide fraction of camelina mucilage, whose structural features might also be due to a partial degradation of polysaccharide chains, which occurs during the extraction process at temperatures higher than 100 °C [5,28].

### 3.2. CCP Rheological Properties

**Figure 1:**
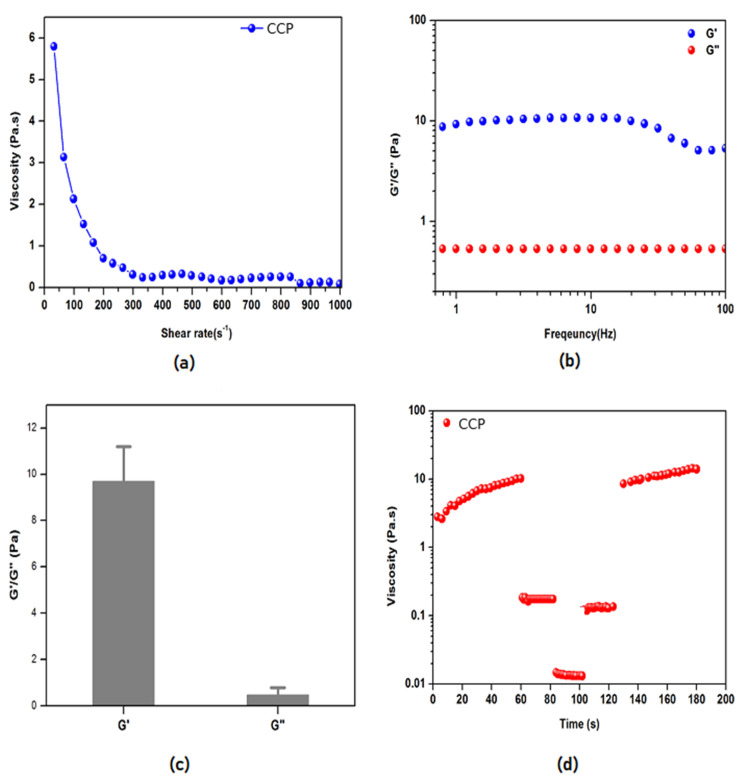
Rheological studies on the viscoelastic properties of CCP. (**a**) Viscosity measurements for increasing the shear rate of CCP. (**b**) Frequency-dependent oscillatory rheology (0.1–100 Hz) of CCP featuring a predominant solid-elastic behavior (G’) as compared to the viscous component (G”), (**c**) Average values of storage (G’—blue color) and loss (G”—red color) moduli obtained from frequency-sweep tests. (**d**) Thixotropy test of CCP solution showing its space-filling propensity.

The viscoelastic properties of CCP were evaluated by using shear-rate rheology. Firstly, we performed viscosity tests in order to assess the Newtonian/non-Newtonian flow behavior of CCP (Figure 1a). CCP showed non-Newtonian behavior through a decrease in viscosity as the shear-rate increased. The high viscosity of the sample (5.9 Pa·s) at low shear rates (0.001 s^−1^) provided insights for its long-term stability in the application of hydro-colloidal systems and could be attributed to the intermolecular interactions among protein and polysaccharide molecules, which would result in the formation of entangled networks, similar to those observed for Camelina seed gum by Li et al. [29]. On the other hand, the reduction in viscosity at a high shear rate (1000 s^−1^) highlighted its shear-thinning propensity, which typical of hydrogel-like materials and was also observed in other food-derived materials (i.e., soybean and lupine peptides) [30].

CCP mechanical properties were evaluated by measuring the storage (G’) and loss (G”) moduli using oscillatory shear rheological experiments (Figure 1b). G’ reflects the stiffness, and G” represents the energy dissipated during the oscillatory test and correlated with the liquid-like response of the sample. The ratio between G’ and G” provided insights into the viscoelastic profile, i.e., whether a material behaved as an elastic solid (G’ > G”) or a viscous liquid (G’ < G”) [31]. In Figure 1b, G’ (in blue) and G” (in red) moduli trends of CCP showed typical hydrogel-like profiles, featuring predominant solid-elastic behavior (G’) as compared to the viscous component (G”). Throughout the tested frequency range (0.1–100 Hz) at a fixed strain (0.5%), CCP displayed G’ and G” mean values of 9.8 Pa and 0.6 Pa, respectively (Figure 1c), in agreement with the data obtained for camelina gum fibers by Li et al., (2016) [29].

Lastly, CCP thixotropy (i.e., its propensity to recover the initial viscosity after shear-rate changing, by simulating an injection) was evaluated. CCP exhibited fast recovery after injection simulation through a series of constant shear rate tests (see Materials and Methods for further details) (Figure 1d). This fast viscosity recovery hinted to its space-filling propensity, which allowed CCP gel-like structure to break and then to recover its structure when the stress was removed.

### 3.3. CCP Stabilizing Effect on Bioaccessibility of Encapsulated MCE Polyphenols

Considering the above results and the effect of CCP in prolonging MCE shelf-life [17], the improvement of MCE phytocomplex bioaccessibility in the ingredients obtained by the encapsulation of MCE with CCP 50% (MCE-CCP50) and CCP 75% (MCE-CCP75) was investigated.

Thirteen different marker compounds selected in MCE were monitored in MCE-CCP50 and MCE-CCP75 at different time intervals during the in vitro simulated digestion process, as reported in Table 1 and Table 2, respectively.

The bioaccessibility index was calculated for each of anthocyanin (cyanidin-3-*O*-glucoside, perlagonidin-3-*O*-glucoside, and peonidin-3-*O*-glucoside), flavonol (myricetin-7-*O*-hexoside, isorhamnetin-3,7-di-*O*-hexoside, quere-cetin-7-*O*-*p*-cumaroylhexoside, quercetin-7-*O*-glucoside, kaempferol-7-*O*-(6″-*O*-malonyl)-hexoside, isorhamnetin-7-*O*-rutinoside, isorhamnetin-3-*O*-hexoside, luteolin-7-*O*-glucoside, kaempferol-3-*O*-hexosyl-7-*O*-glucuronilhexoside), and hydroxycinnamic acid (ferulic acid derivative) as the percentage of soluble compound detected in the collected digestive fractions in comparison with that expected following the gradual dilution occurring during the static in vitro digestion procedure [8].

Regarding MCE-CCP50 (Table 1), during the oral and gastric phases, the bioaccessibility indexes for kaempferol, myricetin, and quercetin derivatives were higher than expected, and the following observed reductions during the monitoring period were probably attributable to the dilution occurring during the digestion. The same trend was observed in the MCE-CCP75 fraction (Table 2), suggesting that the release of polyphenols from both the ingredients was very fast during the oral phase and at the beginning of the gastric phase, as showed by the highest bioaccessibility index registered after 15 min of digestion. Conversely, during the intestinal phase, all the registered bioaccessibility indexes were lower than those expected on dilution basis for both the digested ingredients, but still detectable, differently from what previously observed for MCE [8]; therefore, this behavior could suggest that CCP effectively improved MCE bioaccessibility.

The flavonols’ release trend registered for CCP-based ingredients agreed with that reported for polysaccharide-based hydrogels [32], probably due to CCP matrix gradual swelling and erosion during the digestion process.

The only exception was registered for quercetin-7-*O*-*p*-cumaroylhexoside: during MCE-CCP50 digestion, its bioaccessibility index was 93.97% after the oral phase, it de-creased to 70.28% at the beginning of the gastric phase, and then was 91.34% after 2 h, suggesting that the release rate of this compound was higher than those registered for the other markers and balanced the gradual dilution occurring during the digestion process.

Note: Following the INFOGEST protocol, samples were diluted 1:1 (*v*/*v*) at the beginning of each digestion phase; thus, the expected bioaccessibility index was based on this dilution factor.

**Figure 2:**
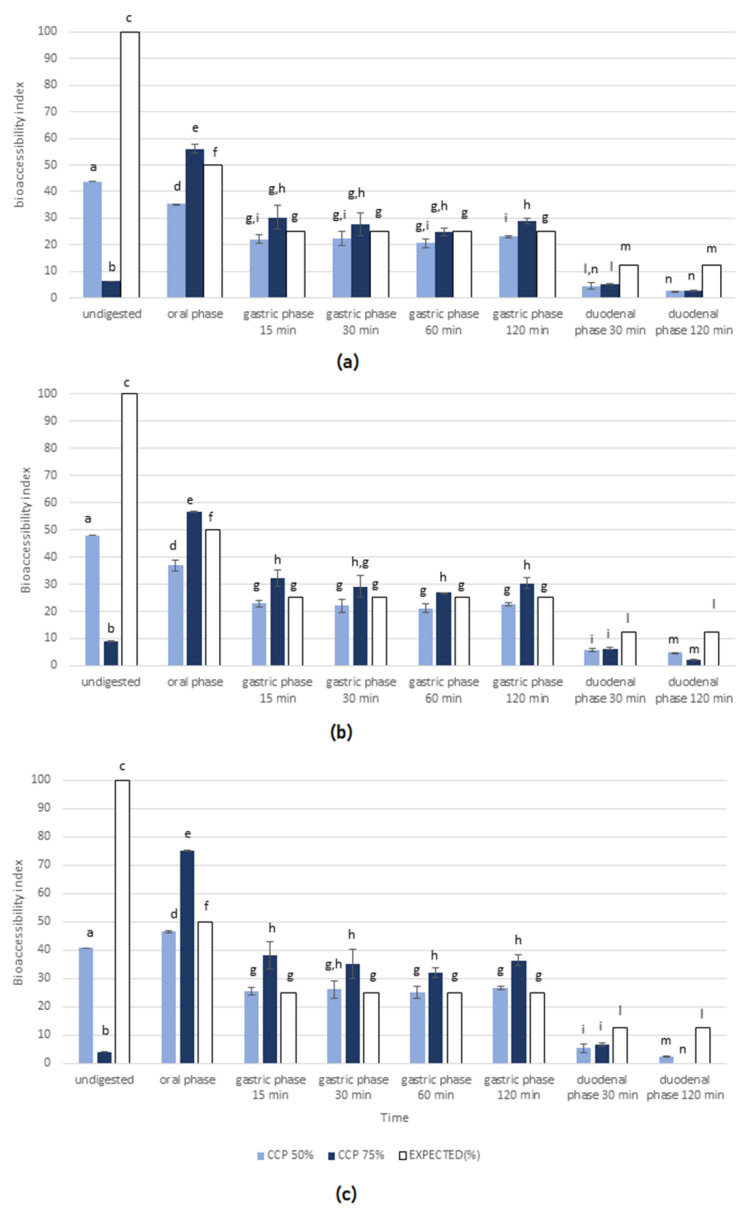
Experimental bioaccessibility index. Values registered for (**a**) cyanidin-3-*O*-glucoside, (**b**) perlagonidin-3-*O*-glucoside, and (**c**) peonidin-3-*O*-glucoside in MCE-CCP50 (light blue bars) and MCE-CCP75 (dark blue bars) at different time points. White bars represented the expected bioaccessibility index on a gradual dilution basis (occurring during the in vitro digestion process). Different lowercase letters indicate significant differences for each compound (*p* < 0.05).

Conversely, the quercetin-7-*O*-*p*-cumaroylhexoside release trend from MCE-CCP75 was slower during the gastric phase, and its bioaccessibility index was still 53.4% after 2 h under gastric conditions, and it did not balance the dilution factor. Among MCE polyphenols, anthocyanins were the most abundant and representative compounds known for their easy degradation [8]. Various delivery systems were tested to improve their bioaccessibility [9,33,34,35,36]. The use of CCP strongly improved the bioaccessibility index for all the anthocyanins present in MCE (Figure 2a–c); in fact, in undigested samples, only the unbound fraction was soluble and bioaccessible, and the registered bioaccessibility index was different according to the anthocyanin and the ingredient content, ranging from 40.68% to 47.93% in MCE-CCP50 and from 3.95% to 9.07% in MCE-CCP75.

After the oral phase, the three anthocyanins were quickly released and the bioaccessibility index was strongly improved; in particular, in MCE-CCP75, cyanidin-3-*O*-glucoside, perlagonid-in-3-*O*-glucoside, and peonidin-3-*O*-glucoside, increased from 6.34%, 9.07%, and 3.95% to 56.09%, 56.77%, and 75.24% respectively. During the gastric phase, almost all the anthocyanin content was already bioaccessible after 15 min in both the fractions. Under gastric acidic environment, anthocyanins were in flavylium cation form and thus relatively stable, as evident from the constant bioaccessible amount until the end of gastric phase. This behavior was already reported in literature by Norkaew et al. [23] and Huang and Zhou [37], who investigated the bioaccessibility of the anthocyanin fraction from black rice encapsulated in two different Arabic gum-based delivery systems. The release rates of these two ingredients were higher during oral and gastric phases due to the rapid degradation of the carrier by α-amylase and pepsin, as probably occurred for CCP, whose released mechanism was based on swelling and erosion according to our tests. Differently from the Arabic gum-based delivery systems, for which during the intestinal phase, the color turned to a dark blue–green in a few minutes due to the pH value change (responsible for anthocyanins degradation to colorless carbinol pseudobase, which in turn degraded to protocatechuic acid and phloroglucinaldehyde) [23,37], the anthocyanins released from MCE-CCP ingredients were still detectable and quantifiable, and the intestinal simulated fluid was pale pink until the end of digestion process. This could suggest that at the intestinal level, CCP was present in a fully hydrated form, which created a viscous matrix able to affect the polyphenols’ diffusion and thus prevent their degradation [12].

Overall, the effects of vegetable carrier agents such as polysaccharides or gums on polyphenols during digestion have been widely investigated [22,38]; and by preserving these compounds from degradation and enhancing their solubility in the intestinal fluids, their bioprotective effects could be maintained, or sometimes enhanced [39].

The results of CCP BS-binding activity at the different tested concentrations are summarized in Table 3. Cholestyramine (10 mg/mL) totally bound 10 mM bile salts, in agreement with literature data [25,40]. Conversely, when CCP was tested at the concentrations used in the ingredients (0.5 mg and 0.75 mg/mL), CCP was not able to trap bile salts, and therefore, the purified polysaccharide fraction could not exert any hypocholesterolemic activity; conversely, at the highest concentrations of 2.5–10 mg/mL, its activity quickly increased, reaching 100%. No significant difference was detected among the four primary bile salts (tested at the same concentration used in the mixture) and their mixture (*p* < 0.05).

Considering that the experimental setup required a centrifugation step to isolate un-bound BS [39], the high activity registered for CCP in the concentration range 2.5–10 mg/mL could be attributed to BS absorption, and these results are similar to those obtained by Gomez, Singh, Acharya, Jayaprakasha, and Patil [41], who investigated the BS binding activity of 3 g/mL of fresh garnet stem leaf powder under the same conditions. However, based on CCP extraction yield [17], 5 mg/mL of the purified polysaccharide fraction corresponded to 50 mg of crude cake; therefore, its binding activity should be considered far higher than the activity reported in literature for other matrices, which were usually crude flours or dietary-fiber-enriched food ingredients [38,39,41].

### 3.4. Bile Salts Binding Capacity of CCP

Another goal of this study was to investigate a putative functional property of the CCP-based ingredient by assessing the relationships between the CCP’s structural and rheological properties and its capacity to retain BS during in vitro simulated digestion.

Soluble and insoluble polysaccharides were reported to interact with bile salts, pre-venting their reabsorption and promoting their transit to the colon, thereby increasing the hepatic synthesis of primary bile acids and reducing serum cholesterol synthesis [39].

**Figure 3:**
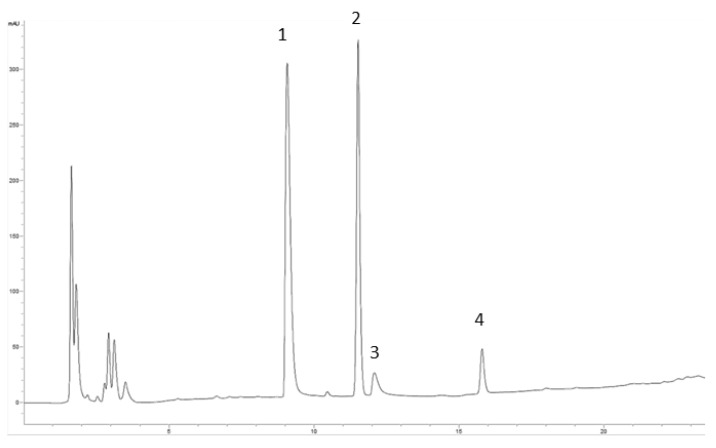
Chromatographic profile of BS mixture in SIF tested at 10 mM final concentration, registered at 208 nm. (**1**) NaTC, (**2**) NaGC, (**3**) NaTCDC, (**4**) NaGCDC.

CCP BS-binding ability was tested at five different concentrations in the range 0.5–10 mg/mL and compared with that of cholesteryramine, a known synthetic bile acid sequestrant [25,40]. An HPLC method that had been previously setup and validated was applied, and the quantification was performed using the external standard method [20]. The calibration curve constructed for NaTC, NaGC, NaTCDC, and NaGCDC had R^2^ > 0.990, and in Figure 3, the profile of the standard mixture is reported.

## 4. Conclusions

CCP was the alcohol-insoluble polysaccharide fraction previously isolated from camelina cake, the main by-product generated from camelina oil production, able to pro-long the shelf life of an MCE-based ingredient when used at 50% and 75% (*w*/*w*). The structural and rheological properties of CCP were deeply investigated in this work, confirming that its carbohydrate fraction is mainly composed by neutral polysaccharides with a broad molecular weight distribution around 139.749 × 103 g/mol. Moreover, CCP showed a typical hydrogel-like profile, resulting in the formation of an entangled network of proteins and polysaccharides with a thixotropic feature. This property could gain it attention in the food or food supplement field, as it could allow the controlled delivery of bioactive compounds.

Furthermore, these rheological properties could justify the results obtained from in vitro digestion of the two MCE-CCP50 and MCE-CCP75 ingredients. In fact, the bioaccessibility index registered for selected polyphenols in MCE highlighted that CCP, at both 50% and 75%, allowed the gradual delivery of such compounds during oral and gastric phases by a swelling mechanism, which was already known of by a composite gel obtained from the interaction of lotus root extract and whey proteins.

Considering CCP’s structural and rheological features and the results obtained from solid-state stability and bioaccessibility studies performed on MCE-CCP50 and MCE-CCP75, it could be concluded that CCP can supply a protective barrier for MCE polyphenols, increasing their storage stability and bioaccessibility. Moreover, CCP, by improving anthocyanins’ stability and keeping constant their concentrations during the digestion process, is supposed to preserve the antioxidant potency of MCE until the intestine.

Therefore, since the ingredient containing CCP 75% represents a valuable solution for stabilizing MCE polyphenols and enhancing their bioaccessibility, hypoglycemic and anti-glycative in vitro tests will be carried out after digestion in order to fully characterize the potential efficacy of this final ingredient in the prevention of chronic and age-related disease risk factors.

## Figures and Tables

**Table 1:** Bioaccessibility index registered for each selected Moradyn Cob Extract (MCE) polyphenol during in vitro digestion of MCE-CCP50.

	Bioaccessibility Index (%)						
Compound	Oral Phase	Gastric Phase (15′)	Gastric Phase (30′)	Gastric Phase (1 h)	Gastric Phase (2 h)	Duodenal Phase (30′)	Duodenal Phase (2 h)
Expected	50	25	25	25	25	12.5	12.5
Cyanidin-3-*O*-glucoside	35.36 ± 0.06	22.18 ± 1.81	22.33 ± 2.59	20.59 ± 1.56	23.12 ± 0.48	4.58 ± 1.25	2.72 ± 0.01
Perlagonidin-3-*O*-glucoside	39.26 ± 2.11	24.35 ± 1.37	23.49 ± 2.54	22.53 ± 1.63	24.12 ± 0.78	6.08 ± 0.62	5.18 ± 0.03
Peonidin-3-*O*-glucoside	48.96 ± 0.48	26.94 ± 1.41	27.45 ± 3.18	26.31 ± 2.26	28.04 ± 0.67	5.53 ± 1.69	2.60 ± 0.26
Ferulic acid derivative	44.87 ± 1.95	21.36 ± 0.13	22.48 ± 1.79	25.38 ± 2.12	23.41 ± 2.29	n.d.	n.d.
Myricetin-7-*O*-hexoside	33.25 ± 2.85	21.05 ± 0.23	24.55 ± 0.64	23.28 ± 0.28	24.46 ± 3.94	12.14 ± 1.13	n.d.
Isorhamnetin-3,7-di-*O*-hexoside	41.82 ± 2.84	23.29 ± 0.01	23.37 ± 0.07	26.78 ± 1.25	24.22 ± 3.34	11.45 ± 1.99	3.66 ± 1.04
Quercetin-7-*O*-*p*-cumaroylhexoside	93.07 ± 1.95	70.28 ± 7.67	79.11 ± 0.91	90.27 ± 3.62	91.34 ± 10.61	33.49 ± 7.23	35.42 ± 4.88
Quercetin-7-*O*-glucoside	64.09 ± 0.66	36.02 ± 2.17	35.97 ± 1.12	39.46 ± 4.00	35.88 ± 1.15	13.80 ± 2.96	10.30 ± 1.86
Kaempferol-7-*O*-(6″-*O*-malonyl)-hexoside	62.69 ± 0.69	32.02 ± 3.80	34.40 ± 1.56	33.68 ± 1.56	33.74 ± 0.40	12.96 ± 3.55	10.48 ± 1.68
Isorhamnetin-7-*O*-rutinoside	59.98 ± 0.46	30.23 ± 0.17	31.81 ± 0.05	51.04 ± 10.65	39.32 ± 6.49	13.11 ± 2.75	14.63 ± 1.87
Isorhamnetin-3-*O*-hexoside	57.57 ± 1.47	25.80 ± 0.82	27.55 ± 1.00	27.86 ± 3.70	27.36 ± 1.53	11.63 ± 3.02	11.64 ± 3.02
Luteolin-7-*O*-glucoside	66.52 ± 1.56	21.48 ± 0.54	23.24 ± 0.19	27.02 ± 0.47	25.86 ± 2.72	10.20 ± 2.69	11.83 ± 1.76
Kaempferol-3-*O*-hexosyl-7-*O*-glucuronilhexoside	66.34 ± 1.85	36.71 ± 1.62	34.68 ± 0.74	35.85 ± 2.17	35.22 ± 2.57	20.18 ± 3.67	19.17 ± 3.45

Note: Following INFOGEST protocol, samples were diluted 1:1 (*v*/*v*) at the beginning of each digestion phase; thus, the expected bioaccessibility index was based on this dilution factor. n.d.: not determined.

**Table 2:** Bioaccessibility index registered for each selected Moradyn Cob Extract (MCE) polyphenol during in vitro digestion of MCE-CCP75.

	Bioaccessibility Index (%)						
Compound	Oral Phase	Gastric Phase (15′)	Gastric Phase (30′)	Gastric Phase (1 h)	Gastric Phase (2 h)	Duodenal Phase (30′)	Duodenal Phase (2 h)
Expected	50	25	25	25	25	12.5	12.5
Cyanidin-3-*O*-glucoside	56.09 ± 1.77	30.28 ± 4.40	27.75 ± 4.34	24.83 ± 1.49	28.83 ± 0.96	5.35 ± 0.08	2.78 ± 0.07
Perlagonidin-3-*O*-glucoside	56.77 ± 0.13	32.11 ± 3.10	29.19 ± 3.86	26.83 ± 0.03	30.33 ± 2.10	6.34 ± 0.53	2.24 ± 0.19
Peonidin-3-*O*-glucoside	75.24 ± 0.07	38.32 ± 4.80	35.03 ± 5.10	31.98 ± 1.65	36.37 ± 1.82	6.59 ± 0.75	n.d.
Ferulic acid derivative	63.59 ± 0.32	32.02 ± 2.67	24.14 ± 4.41	18.12 ± 1.02	20.06 ± 0.01	n.d.	n.d.
Myricetin-7-*O*-hexoside	46.49 ± 1.89	30.51 ± 1.30	30.76 ± 1.46	23.83 ± 1.04	24.51 ± 0.18	13.06 ± 0.33	2.14 ± 0.82
Isorhamnetin-3,7-di-*O*-hexoside	63.54 ± 1.54	29.06 ± 1.34	30.94 ± 6.35	23.87 ± 1.92	25.05 ± 1.28	16.27 ± 3.38	3.51 ± 0.31
Quercetin-7-*O*-p-cumaroylhexoside	89.89 ± 0.62	57.80 ± 4.00	60.58 ± 2.20	49.51 ± 0.29	53.40 ± 3.28	26.03 ± 5.83	23.50 ± 2.63
Quercetin-7-*O*-glucoside	n.d.	71.07 ± 2.74	66.77 ± 5.37	61.71 ± 0.43	67.24 ± 0.32	24.95 ± 1.86	7.06 ± 2.99
Kaempferol-7-*O*-(6″-*O*-malonyl)-hexoside	70.85 ± 1.43	39.86 ± 4.29	37.27 ± 8.36	30.89 ± 1.02	33.97 ± 1.40	13.27 ± 1.33	6.85 ± 1.72
Isorhamnetin-7-*O*-rutinoside	67.80 ± 1.94	45.17 ± 5.16	35.40 ± 0.35	32.84 ± 0.61	44.30 ± 9.83	26.23 ± 4.00	20.00 ± 2.97
Isorhamnetin-3-*O*-hexoside	64.63 ± 1.32	30.41 ± 3.53	27.60 ± 4.89	23.92 ± 1.05	26.81 ± 1.15	12.22 ± 0.90	10.51 ± 0.28
Luteolin-7-*O*-glucoside	55.98 ± 1.82	26.90 ± 2.16	29.86 ± 10.74	20.93 ± 1.70	25.72 ± 2.94	12.45 ± 1.52	10.62 ± 0.64
Kaempferol-3-*O*-hexosyl-7-*O*-glucuronilhexoside	81.84 ± 2.97	40.21 ± 3.39	31.82 ± 12.33	29.30 ± 2.68	33.13 ± 2.32	27.88 ± 2.57	24.96 ± 1.72

Note: Following the INFOGEST protocol, samples were diluted 1:1 (*v*/*v*) at the beginning of each digestion phase; thus, the expected bioaccessibility index was based on this dilution factor. n. d.: not determined.

**Table 3:** Camelina Cake Polysaccharide (CCP) binding capacity (%) calculated for each primary bile salt (BS) or for the BS mixture (35% NaTC, 35% NaGC, 15% NaTCDC and 15% NaGCDC).

Bile Acid Binding Activity (%)					
CCP (mg/mL)	NaTC	NaGC	NaTCDC	NaGCDC	BS Mixture
0.50	N.A.	N.A.	N.A.	N.A.	N.A.
0.75	N.A.	N.A.	N.A.	N.A.	N.A.
2.50	38.21 ± 2.29	40.26 ± 2.25	41.27 ± 0.04	41.27 ± 2.13	39.88 ± 0.72
5.00	100.00 ± 0.13	100.00 ± 0.08	100.00 ± 0.01	100.00 ± 0.01	100.00 ± 2.97
10.00	100.00 ± 0.01	100.00 ± 0.07	100.00 ± 0.01	100.00 ± 0.01	100.00 ± 1.28

N.A. indicates no activity.

## Data Availability

The data presented in this study are available in the present article.
